# Increased Symmetric Dimethylarginine Level Is Associated with Worse Hospital Outcomes through Altered Left Ventricular Ejection Fraction in Patients with Acute Myocardial Infarction

**DOI:** 10.1371/journal.pone.0169979

**Published:** 2017-01-26

**Authors:** Julie Lorin, Jean-Claude Guilland, Karim Stamboul, Charles Guenancia, Yves Cottin, Luc Rochette, Catherine Vergely, Marianne Zeller

**Affiliations:** 1 Laboratory of Cardiometabolic Physiopathology and Pharmacology, INSERM UMR 866, UFR Sciences de Santé, Université de Bourgogne, Dijon, France; 2 Service de Cardiologie, CHU Dijon, France; University of Bologna, ITALY

## Abstract

**Objectives:**

We aimed to investigate whether SDMA- symmetric dimethylarginine -the symmetrical stereoisomer of ADMA- might be a marker of left ventricular function in AMI.

**Background:**

Asymmetric dimethylarginine (ADMA) has been implicated in the prognosis after acute myocardial infarction (AMI) and heart failure (HF).

**Methods:**

Cross sectional prospective study from 487 consecutive patients hospitalized <24 hours after AMI. Patients with HF on admission were excluded. Serum levels of ADMA, SDMA and L-arginine were determined using HPLC. Glomerular filtration rate (eGFR) was estimated based on creatinine levels. Outcomes were in-hospital severe HF, as defined by Killip class >2, and death.

**Results:**

Patients were analysed based on SDMA tertiles. Sex, diabetes, dyslipidemia, and prior MI were similar for all tertiles. In contrast, age and hypertension increased across the tertiles (p<0.001). From the first to the last tertile, GRACE risk score was elevated while LVEF and eGFR was reduced. The rate of severe HF and death were gradually increased across the SDMA tertiles (from 0.6% to 7.4%, p = 0.006 and from 0.6% to 5.0%, p = 0.034, respectively). Backward logistic multivariate analysis showed that SDMA was an independent estimate of developing severe HF, even when adjusted for confounding (OR(95%CI): 8.2(3.0–22.5), p<0.001). Further, SDMA was associated with mortality, even after adjustment for GRACE risk score (OR(95%CI): 4.56(1.34–15.52), p = 0.015).

**Conclusions:**

Our study showed for the first time that SDMA is associated with hospital outcomes, through altered LVEF and may have biological activity beyond renal function.

## Introduction

Coronary artery disease (CAD) including acute myocardial infarction (MI) is the most frequent cause of altered Left Ventricular Ejection Fraction (LVEF) and Heart Failure (HF). Conversely, HF is a frequent complication of acute MI and significantly worsens the prognosis of patients with CAD. Given the strong association of acute MI and HF, it is important to understand the underlying mechanisms of HF in patients with acute MI.

Impaired nitric oxide (NO) bioavailability is involved in the pathogenesis and progression of CAD. Moreover, in patients with chronic HF, accumulation of methylated arginine metabolites has been associated with disease progression [1]. Asymmetric dimethylarginine (ADMA), as a methylated product of L-arginine, may compete with L-arginine as the substrate for the nitric oxide synthases (NOS) or inhibit NOS phosphorylation and therefore decrease NO production [2]. Over the last decades, it has emerged as a novel cardiovascular risk factor in the setting of endothelial dysfunction including type 2 diabetes, hypertension, CAD, HF and end stage renal disease [3,4].Moreover, in recent works we have suggested the specific link between ADMA and HDL levels [5].

Much less is known about the biological role of an alternative methylation product of L-arginine, namely symmetric dimethylarginine (SDMA). Although structural isomer of ADMA, SDMA is not a direct competitive inhibitor of NOS, but could interfere with L-arginine uptake into the cells via the y+ class of cationic amino acid transporters (CAT) [6]. SDMA is mainly cleared by the renal route and its circulating levels are elevated in chronic kidney disease. SDMA has been initially considered as a powerful marker of renal function [7]. Recent lines of evidence showed that SDMA could not be simply yet another uremic toxin, but also a mediator with pathophysiological relevance as an early diagnostic for detrimental cardiovascular outcomes [8,9]. Notably, in patients with acute MI, elevated SDMA levels are strong predictors of late cardiac events, beyond chronic kidney disease [10]. Moreover, in patients with chronic systolic HF, SDMA levels were associated with the presence of LV dysfunction, suggesting a potential role of SDMA in the pathophysiological of HF [1].

In a subgroup analysis from a large prospective study in acute MI patients [5], we evaluated the relationship between circulating levels of dimethylarginines, in particular SDMA, with renal function and LVEF.

## Methods

### Study subjects

All the consecutive patients aged >18 years and hospitalized <24 hours after symptom onset for acute MI in the Coronary Care Unit of Dijon University Hospital from 1^st^ January 2011 to 30^th^ June 2012 were included. Patients with relevant co-morbidities (infection, autoimmune disorders and cancers) or admitted with heart failure were excluded from the study. MI was defined by an increase in serum troponin Ic [> upper limit of the hospital normal (ULN) range: 0.1 μg/L] associated with symptoms of ischemia and/or typical ECG signs. ST-segment elevation MI (STEMI) was defined as chest pain lasting for ≥20min with typical ECG changes including ≥1mV ST segment elevation in two or more limb leads or ≥2mV in two or more contiguous precordial leads. The study was approved by the Consultative Committee of Protection of Persons in Biomedical Research of Burgundy and conducted in accordance with Declaration of Helsinki. All subjects gave their written consent to participate in the study.

### Data collection

Data on demographics, risk factors [history of hypertension, diabetes, dyslipidemia, body mass index (BMI)], chronic treatments and prior MI were prospectively collected. Chronic kidney disease was defined based on the presence of kidney damage or glomerular filtration rate (eGFR<60 mL/min per 1.73 m2) for 3 months, irrespective of cause[11].

History of HF was defined as previous hospital admission with diagnosis of HF or documented clinical symptoms of HF. Killip class was classified as follow: Killip I: no clinical signs of HF; Killip class II: rales or crackles < 50% lung field; Killip class III frank acute pulmonary oedema; Killip class IV: cardiogenic shock. In-hospital severe HF was defined by Killip class >II. The Global Registry of Acute Coronary Event (GRACE) risk score was calculated for each patient with admission variables including age, heart rate, serum creatinine, systolic blood pressure, Killip class, cardiac arrest, ST-segment deviation, and cardiac markers (http://www.outcomes-umassmed.org/grace/). Echocardiography was performed at 2±1 days with the Simpson method to assess LVEF. Altered LVEF was defined as LVEF ≤ 40% for more clinical relevance.

### Biological data

Blood samples were drawn on admission (Median time from symptom onset to blood sampling: 16 (8–20) hours), as previously described [5]. Creatinine levels were measured on a Vitros 950 analyzer (Ortho Clinical Diagnostics) and the glomerular filtration rate (eGFR) was estimated on the Chronic Kidney Disease (CKD) EPI formula [12]. Normal or weakly impaired renal function, moderate and severe renal dysfunction were defined as eGFR > 60 mL/min, 30–60 mL/min and < 30 mL/min respectively. Plasma troponin Ic peak was assessed by sampling every eight hours during the first two days after admission (Dimension Vista Intelligent Lab System, Siemens).

### Dimethylarginines and L-arginine analysis

Samples were allowed to clot at room temperature for 30 minutes and centrifuged at 2500 rpm for 10 minutes at 4°C. The serum was kept frozen at -80°C until analysis. As described in detail previously [5], L-arginine, ADMA, and SDMA, were measured by high performance liquid chromatography (HPLC) [13,14].

### Statistical analysis

Continuous data are presented as median [IQR (inter quartile range)] or mean ± SD, as appropriate or as proportion. For continuous variables, a Kolmogorov-Smirnov analysis was performed to test for normality. Non normal variables were log-transformed before entering into the analysis (i.e. SDMA, NtproBNP, ADMA and Larginine). To compare the data between 2 groups, the Mann-Whitney Rank Sum test or student’s t test was performed and a 1-way ANOVA or by Kruskal-Wallis 1-way analysis, as appropriate, was performed for 3 groups comparisons. Dichotomous variables were compared by Chi square tests.

Multiple linear regression analysis was performed with eGFR as a dependent variable with age and SDMA as covariates.

No data were available on the relationship between SDMA levels and HF in patients with acute MI. Hence, we have empirically calculated our sample size, based on the number of patients to include for an expected number of 20 events (severe heart failure) chosen as the minimal number of events for adequate statistical performance and yielding a study population at ≈ 500 subjects with MI. Logistic regression analysis was further performed to estimate the variables associated with the risk of developing severe HF during the hospital stay. Only variables that were significant by univariate analyses (p<0.01) were entered into the multivariate models, with an exclusion cut-off at 2%. The first model included LVEF, eGFR, and Nt-proBNP. A second model included LVEF, eGFR and SDMA (instead of Nt-proBNP). ROC curve analysis was further performed for each model and AUC were used to compare the discriminative value for the 2 models.

Another model of backward logistic regression analysis was built to assess the association between SDMA and in-hospital mortality. NtproBNP and GRACE risk score were included as covariates, in addition to SDMA, with the same exclusion cutoff. All the analyses were performed using the SPSS 12.0 software package (IBM Inc, USA).

## Results

### Baseline clinical and biological variables

The characteristics of the 487 patients classified by SDMA tertiles are shown in Table 1. Sex, diabetes, dyslipidemia, and prior MI were similar for all tertiles. In contrast, mean age and proportion of patients with hypertension increased across the tertiles (^*p*^<0.001). Anterior location and hemodynamic parameters on admission did not differ significantly. From the first to the last tertile, GRACE risk score was elevated and LVEF was reduced. Rate of PCI was decreased across SDMA tertiles.

**Table 1:** Presenting characteristics (n(%), median (IQR) or mean ± SD).

	SDMA tertiles Median (IQR)range(min-max), μmol/L			
	T10.35(0.31–0.39)0.15–0.42*n* = 163	T20.50(0.46–0.54)0.42–0.59*n* = 163	T30.76(0.66–0.96)0.59–2.48*n* = 161	*p*
Risk factors				
Female	33(20%)	37(23%)	42(26%)	0.474
Age, *years*	59±12	62±13	70±15	<0.001
Diabetes	29 (18%)	28 (17%)	38 (24%)	0.289
Hypertension	69 (42%)	77 (47%)	100 (62%)	0.001
Dyslipidemia	70 (44%)	71 (44%)	67 (42%)	0.889
Current Smoker	64 (39%)	56 (34%)	35 (22%)	0.003
Prior MI	17 (10%)	19 (12%)	26 (16%)	0.249
Chronic kidney disease	0 (0%)	2 (1%)	14 (9%)	<0.001
Clinical data				
STEMI	103 (63%)	82 (50%)	79 (49%)	0.018
Anterior wall location	60 (37%)	66 (41%)	53 (33%)	0.347
Heart rate, *beats/min*	75 [64–85]	74 [64–83]	75 [64–88]	0.842
SBP, *mmHg*	134 [122–160]	140 [117–160]	139 [116–161]	0.961
DBP, *mmHg*	84 [70–96]	80 [70–90]	79 [69–90]	0.026
GRACE risk score	127±26	132±30	147±33	<0.001
LVEF, *%*	55 [50–60]	58 [50–65]	50 [45–60]	0.010
Chronic treatments				
Aspirin	24 (15%)	27 (17%)	42 (26%)	0.020
Betablocker	26 (16%)	40 (25%)	49 (30%)	0.009
Fibrate	9 (6%)	6 (4%)	2 (1%)	0.107
ACE inhibitor	21 (13%)	31 (19%)	30 (19%)	0.259
Statin	35 (22%)	37 (23%)	41 (26%)	0.684
Acute revascularization				
PCI	124 (76%)	109 (67%)	99 (63%)	0.017
Thrombolysis	39 (24%)	43 (26%)	29 (18%)	0.172
CABG	6 (4%)	15 (9%)	11 (7%)	0.130

MI: Myocardial Infarction; CV: Cardiovascular; ACE: angiotensin-converting enzyme; CAD: coronary artery disease; CABG: Coronary Arterial Bypass Graft; SBP: Systolic blood pressure; DBP: Diastolic blood pressure; HR: heart rate; LVEF: left ventricular ejection fraction; PCI: Percutaneous Coronary Intervention; SBP: systolic blood pressure; SDMA: Symmetric dimethylarginine; STEMI: ST segment elevation MI.

Biological data are presented in Table 2. Lipid parameters vary significantly among the 3 groups. A dramatic increase in Nt-proBNP level was observed in the last SDMA tertile (p<0.001). ADMA, L-arginine and homocysteine levels gradually increased across the tertiles.

**Table 2:** Biological data (Median (IQR) or mean ± SD).

	SDMA tertiles Median (IQR)range(min-max), μmol/L			
	T10.35(0.31–0.39)0.15–0.42N = 163	T20.50(0.46–0.54)0.42–0.59N = 163	T30.76(0.66–0.96) 0.59–2.48N = 161	*p*
eGFR, *ml/min/1.73 m^2^*	84±19	80±21	62±26	<0.001
Nt-proBNP, *pg/ml*	252 [79–959]	459 [167–1396]	1343 [322–5249]	<0.001
Homocysteine, *μmol/L*	11 [9–14]	12 [9–16]	14 [11–21]	<0.001
ADMA, *μmol/L*	0.53 [0.38–0.82]	0.57 [0.44–0.94]	0.70 [0.50–1.13]	<0.001
L-arginine, *μmol/L*	86 [70–115]	96 [72–127]	96 [74–128]	0.022
LDL-cholest, *mg/dl*	131±38	130±41	118±45	0.006
HDL-cholest, *mg/dl*	39 [30–47]	40 [33–52]	43 [33–54]	0.002
Total-cholest, *mg/dl*	201±44	202±44	188±51	0.013
Triglycerides, *mg/dl*	121 [89–177]	127 [084–176]	103 [76–151]	0.043
CRP, *mg/L*	4.6 [3.0–10.8]	5.0 [3.0–13.5]	5.6 [3.0–15.0]	0.347
Troponin Ic, peak, *μg/L*	20.0 [6.4–41.0]	19.0 [4.0–41.0]	12.7 [2.9–41.0]	0.163

ADMA: Asymmetric dimethylarginine; CRP: C-reactive protein; eGFR: Estimated Glomerular filtration rate; HDL-C: high density lipoprotein; LDL-C: low density lipoprotein; NT-proBNP: N-terminal Pro-Brain Natriuretic Peptide. SDMA: Symmetric dimethylarginine.

### SDMA and renal function

**Fig 1:**
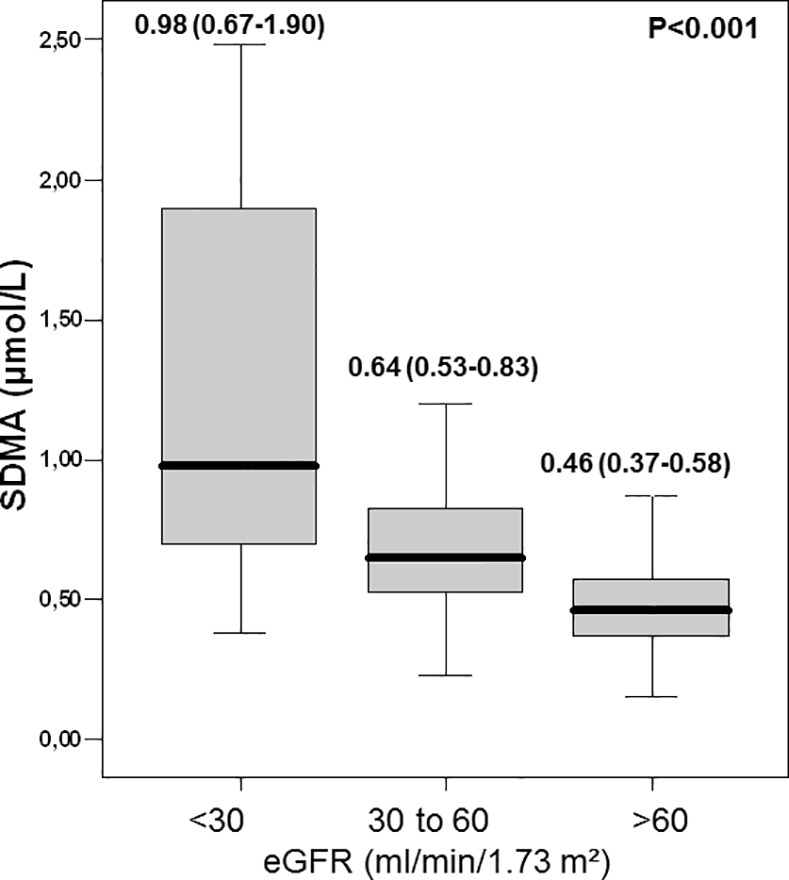
Symmetric dimethylarginine concentrations according to estimated Glomerular Filtration Rate (*p* value for comparison between the tertiles).

Rate of patients with chronic renal failure was almost 10 fold higher in the last tertile than lower tertiles (^*p*^<0.001) (Table 1). Moreover, eGFR markedly decreased across the SDMA tertiles from 84 to 62 ml/min/1.73 m^2^ (Table 2). Given the potential for renal clearance to serve as an important determinant of plasma levels of dimethylated products of L-arginine, in particular for SDMA levels, patients were classified according to their eGFR. SDMA levels showed a strong and gradual relationship with renal filtration rate (Fig 1).

Patients with severely altered renal dysfunction (eGFR <30 ml/min/1.73 m^2^) had markedly higher SDMA levels than patients with normal renal function (≈ +113%, 0.98 vs. 0.46 *μ*mol/L). In contrast, no significant difference was seen for ADMA (0.55(0.43–0.80), 0.58(0.43–1.01), 0.61(0.43–0.89) μmol/L, ^*p*^ = 0.860) or L-arginine levels (86(56–114), 87(69–117), 91(72–125) μmol/L, ^*p*^ = 0.104) in the 3 groups. A significant correlation between between SDMA and eGFR was found (r = -0.499, p<0.001); although less strong, this correlation was also reported in STEMI patients (r = -0.335, p< 0.001). Moreover, by multivariate linear regression analysis, SDMA remained an independent predictor of eGFR (B = -18.0 ± 2.0, ^*p*^<0.001) even after adjustment on age (B = -0.8 ±0.1, ^*p*^<0001).

### SDMA and LVEF

**Fig 2:**
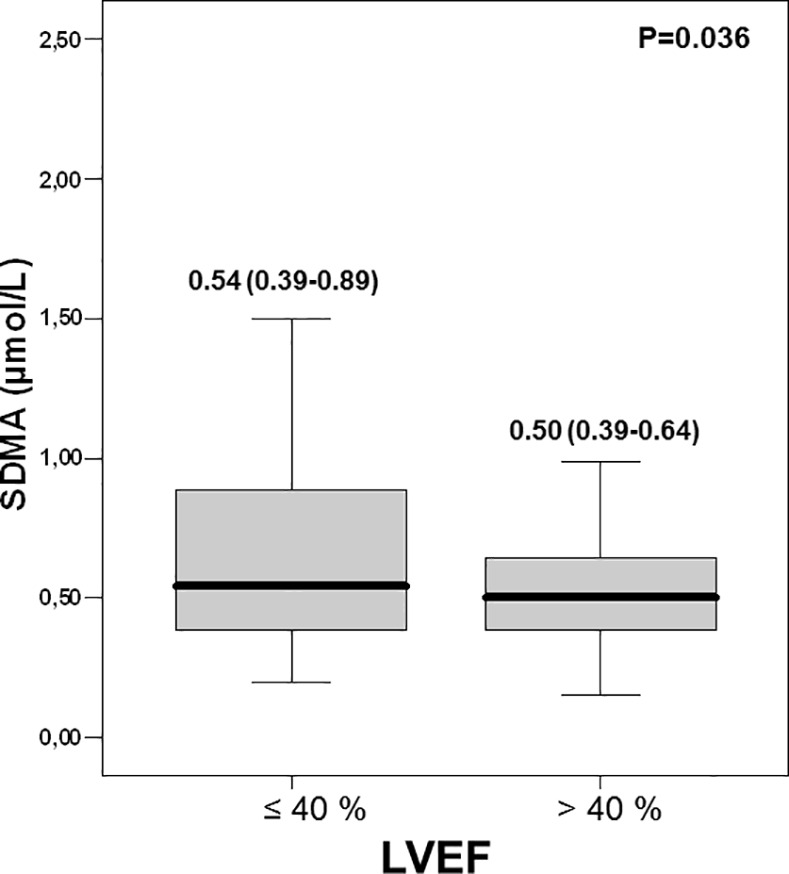
Symmetric dimethylarginine concentrations according to Left Ventricular Ejection Fraction (^*p*^ value for comparison between the 2 groups).

A significant correlation between between SDMA and LVEF was found (r = -0.124, p<0.009); although less strong, this correlation was also reported in STEMI patients (p = 0.011). When patients were classified according to LVEF alteration (≤ 40%) (Fig 2), SDMA was significantly elevated in patients with altered LVEF (0.54 (0.39–0.89) vs. 0.50 (0.39–0.64) μmol/L, *p* = 0.04).

In contrast, there was no difference for the two groups for ADMA and L-arginine levels (0.54 (0.43–0.74) vs.0.56 (0.42–0.87); *p* = 0.41 and (89 (75–117) vs. 89 (70vs. 121) μmol/L; *p* = 0.99, respectively).

### SDMA and in-hospital outcomes

**Fig 3:**
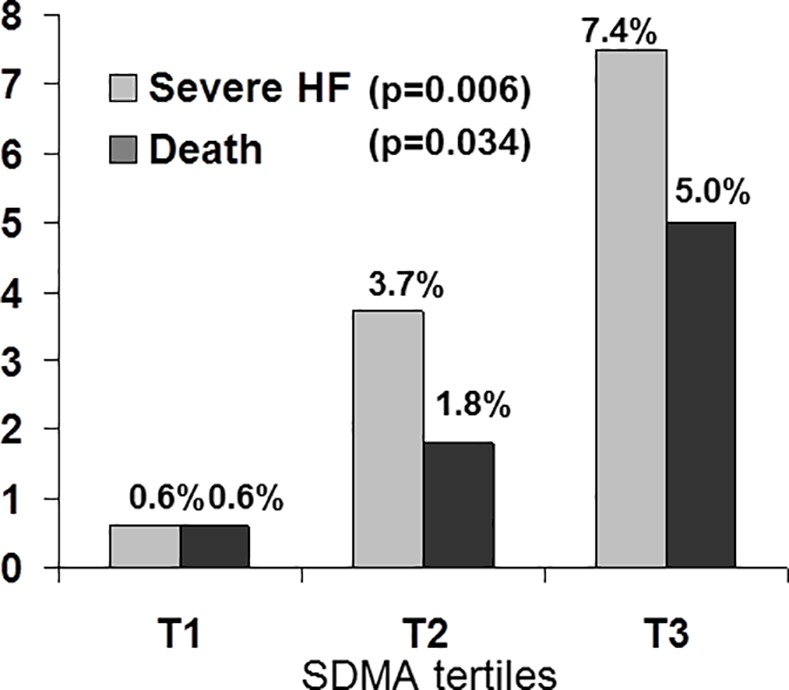
Cardiovascular outcomes according to Symmetric dimethylarginine tertiles values (*p* value for comparison between the tertiles).

Median length of hospital stay was 4(3–4) days. During hospital stay, 19 patients developed severe HF and 12 patients died. The rate of severe HF was similar in STEMI and NSTEMI (5% vs 4%, p = 0.645, respectively). Fig 3 shows the distribution of hospital events across the SDMA tertiles.

Markedly more patients had HF in the higher tertiles of SDMA, with a rate of severe HF more than 10 fold higher in the last tertile when compared to the lowest tertile (p = 0.006). Similarly, there was a gradual increase in mortality across the SDMA tertiles, from 0.6% to 5.0% (*p* = 0.034). Given the strong relationship between SDMA and LVEF, we further tested whether SDMA could estimate the later development of HF during hospital stay. The first model included LVEF, eGFR, and Nt-proBNP. By backward logistic multivariate analysis, Nt-proBNPlevels, in addition to altered LVEF, was an independent estimate of developing severe hospital HF (OR(95%CI): 1.7(1.2–2.3), *p* = 0.002 and 10.7(3.8–30.4), *p*<0.001). ROC curve analysis for the model provided an AUC at 0.860 (SE: 0.047), *p*<0.001. A second model showed that SDMA -as covariate instead of Nt-proBNP- remained an independent predictor of severe HF beyond altered LVEF (OR(95%CI): 8.2(3.0–22.5), *p*<0.001, and 16.8(5.8–48.9), *p*<0.001), respectively). The corresponding AUC was 0.863 (SE: 0.042), *p*<0.001). The comparison of the ROC curves of the 2 models gave similar discrimination performance (^*p*^ = 0.951), further suggesting the value of SDMA as biomarker for the prediction of HF. Sensitivity analyses, based on the location of MI (anterior vs other or unknown location) were further performed. SDMA remained an estimate of HF development in patients with anterior wall location (n = 179(37%)) (OR(95%CI): 26.36(0.93–747,30), p = 0.055) or without anterior wall location (OR(95%CI): 5.61(2.03–15.53), p = 0.001).

Moreover, SDMA was associated with excess risk of hospital mortality, even after adjustment for GRACE risk score (OR(95%CI): 4.56(1.34–15.52), *p* = 0.015, and 1.03(1.00–1.05), *p* = 0.013), respectively).

## Discussion

ADMA has been shown to competitively inhibit the binding of L-arginine to the substrate-binding moiety of NOS and is a strong predictor of CV events in selected population [4]. Moreover, we have recently suggested a link between ADMA and HDL-C levels, which may be due to the modulation of eNOS activity [5]. Less attention has been paid to its structural isomer, namely SDMA, as a biomarker in prospective clinical studies. To the best of our knowledge, this is the first study addressing the role of SDMA in high risk patients. Our large cross sectional prospective study showed that SDMA is a powerful factor associated with alteration of LVEF, beyond its relationship with renal function. Moreover, our findings showed that the circulating levels of this dimethylarginine on admission were predictive for worse outcome, characterized by the development of HF.

### SDMA and renal function

Elimination of SDMA occurs primarily by renal excretion and not surprisingly, we found its levels particularly elevated in patients with acute MI, characterized by frequent impaired renal function. The levels of SDMA were markedly elevated when compared with SDMA levels in healthy individuals from a large community-based cohort using a stable isotope dilution assay [15] [median (IQR), our study: 0.52(0.40–0.71) vs. 0.37(0.32–0.43) μmol/L in Schwedhelm et al]. Our findings on the strong relationship of SDMA serum levels with eGFR are in agreement with both animal experiments and human studies, where SDMA has emerged as an endogenous marker of renal function. In particular, evidence has accumulated from > 2100 patients with CKD or CAD[7], with a correlation coefficient between SDMA and creatinine levels at 0.75 (p<0.001).

### SDMA and outcomes

Although SDMA was usually thought to be functionally inactive, recent works highlighted its predictive value for CV events[16]. However, these works were limited to specific subgroups of CAD patients. Our works extent these findings across the whole spectrum of acute MI. Although SDMA lacks competitive NOS inhibitory activity, all methylated arginine metabolites are thought to inhibit NO synthesis indirectly as L-arginine analogues via blockade of cationic arginine transport–a process shown to be impaired in the setting of HF [17,18]. Moreover, presence of elevated levels SDMA inside the HDL particles may also participate to the noxious vascular effects of the lipoprotein in CKD [9]. Our work is the first to highlight the strong and independent relationship between SDMA, but not ADMA, and altered LV function in acute MI. Moreover, we found that elevated levels of SDMA are strongly associated with the development of HF during hospitalisation, beyond traditional factors including renal impairment. A direct relationship between accumulation of methylated arginine metabolites, including SDMA and altered LV diastolic function has been reported in patients with chronic systolic HF [1]. Moreover, there is strong *in vivo* and *in vitro* evidence for a marked depression of L-arginine transport [17] and endothelial dysfunction in patients with chronic HF. Plasma ADMA levels were also reported elevated in patients with chronic HF, and correlated significantly with New York Heart Association (NYHA) functional class and exercise capacity [19]. In addition, elevated plasma ADMA levels were associated with increased risk of mortality and adverse cardiovascular outcome in patients with chronic HF [20,21]. After an acute ischemic stroke, short term survival decreased significantly with ascending tertiles of SDMA levels at admission [22].

Our findings also found an independent association between SDMA and short term mortality (OR: 4.55(1.34–15.52)), further suggesting a pathophysiological role for this marker. A recent study also showed that SDMA was an independent predictor of all cause and cardiovascular mortality in a large multiethnic population-based cohort [23].

## Limitations

This work, however, suffers the usual limitations of observational, non-randomized studies, and therefore determines association, rather than causal relationships. Other potential factors may influence the prognosis in the setting of acute MI, including delays for invasive strategy, location of chronic total occlusion or multivessel disease[24–26]. These factors were not available in our study and may potentially alter the strength of our findings. However, no data are currently available on the relationship between SDMA and such confounding. Although the origin of circulating dimethylarginines levels remains unclear, endothelial cells may be a major contributor [8]. The involvement of cardiac EC, in conjunction with the pulmonary endothelial cells, is of critical importance in the development of HF because this represents the largest single source of endothelial mediators[27]. In experimental post-MI setting, EC dysfunction has been shown to contribute to the development of HF [28] and impaired prognosis[29]. Dimethylarginines levels in HF have been suggested as a pathophysiologic link between oxygen radical load and impaired vasodilator capacity [29]. Preincubation with SDMA has been shown to stimulate ROS production in isolated monocytes by stimulating Ca^2+^ entry via the SOCs [30] and was associated with an increase of reactive oxygen species in culture endothelial cells [31]. None of these effects were found with ADMA. However, further studies are needed to better understand the underlying biological effects of SDMA.

## Conclusion

Our large prospective study is the first to date showing an association between elevated SDMA and worse hospital outcomes including major surrogate markers in acute MI, such as altered renal function. Our works suggests that such dimethylarginines may probably exert biological activity by other pathways than NOS activity inhibition and at least partly beyond renal function.
